# Spatial Hearing by Bilateral Cochlear Implant Users With Temporal Fine-Structure Processing

**DOI:** 10.3389/fneur.2020.00915

**Published:** 2020-09-30

**Authors:** Sebastián A. Ausili, Martijn J. H. Agterberg, Andreas Engel, Christiane Voelter, Jan Peter Thomas, Stefan Brill, Ad F. M. Snik, Stefan Dazert, A. John Van Opstal, Emmanuel A. M. Mylanus

**Affiliations:** ^1^Department of Biophysics, Radboud University, Donders Institute for Brain, Cognition and Behaviour, Nijmegen, Netherlands; ^2^Department of Otolaryngology, University of Miami, Miami, FL, United States; ^3^Department of Otorhinolaryngology, Radboud University Medical Center, Donders Institute for Brain, Cognition and Behaviour, Nijmegen, Netherlands; ^4^Department of Otorhinolaryngology Head and Neck Surgery, St. Elisabeth-Hospital, Ruhr-University Bochum, Bochum, Germany; ^5^MED-EL, Starnberg, Germany

**Keywords:** sound localization, bilateral cochlear implants, fine-structure, interaural level differences, interaural time differences

## Abstract

Several studies have demonstrated the advantages of the bilateral vs. unilateral cochlear implantation in listeners with bilateral severe to profound hearing loss. However, it remains unclear to what extent bilaterally implanted listeners have access to binaural cues, e.g., accurate processing of interaural timing differences (ITDs) for low-frequency sounds (<1.5 kHz) and interaural level differences (ILDs) for high frequencies (>3 kHz). We tested 25 adult listeners, bilaterally implanted with MED-EL cochlear implant (CI) devices, with and without fine-structure (FS) temporal processing as encoding strategy in the low-frequency channels. In order to assess whether the ability to process binaural cues was affected by fine-structure processing, we performed psychophysical ILD and ITD sensitivity measurements and free-field sound localization experiments. We compared the results of the bilaterally implanted listeners with different numbers of FS channels. All CI listeners demonstrated good sensitivity to ILDs, but relatively poor to ITD cues. Although there was a large variability in performance, some bilateral CI users showed remarkably good localization skills. The FS coding strategy for bilateral CI hearing did not improve fine-structure ITD processing for spatial hearing on a group level. However, some CI listeners were able to exploit weakly informative temporal cues to improve their low-frequency spatial perception.

## Introduction

The research performed in bilateral cochlear implant (CI) listeners clearly showed that they outperform users with a unilateral CI. Apart from improved speech understanding in noise (1–5), sound localization performance in the bilateral CI condition also improved when compared to unilateral CI [e.g., (1, 5, 6)]. However, the benefit of bilateral implantation is not equivalent to binaural hearing, and the performance gap between bilateral CI vs. normal-hearing (NH) listeners is still significant (7, 8). Normal-hearing listeners localize sounds in azimuth with high acuity and precision, thanks to the efficient processing of interaural level differences (ILDs) and interaural timing differences (ITDs). ILDs are extracted from the high-frequency hearing range (>3 kHz), while ITDs are conveyed in the temporal fine structure of low-frequency sounds (<1.5 kHz) (9, 10).

CI technology mainly aimed at improving speech perception. Thus, most available pulse-encoding strategies [e.g., continuous interleaved sampling, or CIS (11)] stimulate the different electrodes with a fixed pulse frequency, in which the current strength of the pulses is modulated by the sound's envelope over the full applicable frequency range. To potentially improve the temporal representation, MED-EL included a CI pulse stimulation strategy that aimed to preserve the low-frequency temporal fine structure of the acoustic signal (fine-structure processing, or FSP). Their algorithm incorporates a zero-crossing analysis in the low-frequency range, after which the coded electrical signal is conveyed to electrode contacts of the CI (i.e., up to four channels in the low-frequency apical turn or up to about 1 kHz according to the coding configuration).

Studies have suggested that bilateral CI listeners benefit from the FSP stimulation strategy in a speech discrimination test in noise (12–16), especially for native speakers of tonal languages (17–19). Dorman et al. (20) studied sound localization in FSP and CIS bilateral users and did not find differences between subgroups. In addition, they observed that low-frequency sounds were poorly localized compared to high-frequency and broadband sounds, suggesting that the bilateral FSP is not adding a benefit for sound localization. However, it is unclear whether this negative finding resulted from a low number of low-frequency fine-structure channels since this was not reported. Recently, Eklöf and Tideholm (21) found that half of the CI listeners with FSP had ITD perception within the physiological range (10/20; mean threshold of 330 ± 250 μs) compared to none in the group without fine-structure (FS) coding. Although some FSP listeners appeared to have ITD sensitivity, this stimulation protocol did not improve their low-frequency sound localization. However, their dichotic stimuli, used for the ITD perceptual tests (500-ms tone pips of 250 Hz with rise and fall times of 100 ms), were not the same as those presented in the free field for sound localization (1.6-s duration speech-shaped sounds or low-pass-filtered music snippets).

Here, we performed two experiments to test sound localization and ILD/ITD processing in experienced bilateral CI users with and without FSP. In order to quantify the effect of the number of bilateral fine-structure channels, we assessed performance for three subgroups: (i) without FSP; (ii) with two bilateral FSP channels; or (iii) with four bilateral FSP channels. In principle, if FSP improves the ITD perception of bilateral CI users by providing reliable cues (21), a larger number of available FSP channels could increase the potential to exploit this cue. We presented different frequency ranges to dissociate ITD and ILD perception with psychoacoustical tasks and free-field sound localization.

## Methods

### Listeners

#### CI Users

Twenty-five bilaterally deaf patients implanted with bilateral CIs (BICI) participated in the experiments. Their ages ranged from 22 to 77 years (53 ± 16.3 years). In the Netherlands, bilateral CIs in adults is not reimbursed. Therefore, in order to acquire bilaterally implanted adult subjects, cooperation was sought with the ENT Clinic of St. Elisabeth-Hospital of Ruhr-University in Bochum in our neighboring country Germany, where reimbursement of bilateral cochlear implantation for adults has been standard care for years. All included subjects had been implanted and were recruited at the University Clinic of Bochum (Germany) and traveled to Radboud University in Nijmegen (the Netherlands) to be assessed in the sophisticated spatial hearing labs at the Radboud University in Nijmegen. All research protocols and informed consent forms were approved by the Medical Ethical Committee in Bochum prior to the start of the experiments. Table 1 shows the ages at test, ages of implantation for each ear, device type, and coding strategy in use. Most participants suffered from a progressive hearing loss. Three patients suffered from an infection (P8: encephalitis at birth; P11: pneumococcal meningitis; and P12: mumps infection), and in one case the etiology of the hearing loss was not known (P9). All CI subjects had bilateral profound hearing loss, with no residual hearing in low frequencies at the time of implantation. Electrode impedance measures were within the normal range, demonstrating normal functioning of CI. Normal electrically evoked compound action potentials (eCAPs) were obtained intraoperatively for all subjects, suggesting good coupling between the electrodes and neural substrate. All electrode insertions were reported to be complete, and subjects were implanted with the same electrode design and length in both ears. To study the effect of FSP, we grouped the patients according to the number of bilateral fine-structure channels: “FS4” refers to listeners with four (the maximum number) FS channels on both sides (*n* = 12); “FS2” to two FS channels on either side (*n* = 8); and “no-FSP” indicates either no FS channel at all or a non-matched low number (e.g., 0 and 1 on the right and left sides, respectively, *n* = 5). The latter group was considered as a control group within the CI users.

**Table 1:** Demographic and general information about subjects.

**Subject**	**Age at test (years)**	**Age of implantation**		**Implant and electrode**		**Audio processor**		**Coding strategy**		**FS channels**	
		**R**	**L**	**R**	**L**	**R**	**L**	**R**	**L**	**R**	**L**
P1	77	71	73	Concerto Flex28	Sonata Standard	Sonnet	Sonnet	FS4HR	FS4HR	4	4
P2	62	58	56	Sonata Standard	Sonata Standard	Opus2	Opus2	FSP	FSP	2	2
P3	43	42	39	Sonata Flex28	Sonata Flex28	Opus2	Sonnet	FS4HR	FS4HR	4	4
P4	29	28	29	Sonata Flex28	Sonata Flex28	Sonnet	Sonnet	FS4HR	FS4HR	4	4
P5	71	64	63	Sonata Standard	Sonata Standard	Opus2	Opus2	FS4HR	FS4HR	4	4
P6	76	69	73	Sonata Flex28	Sonata Standard	Opus2	Opus2	FS4HR	FS4HR	4	4
P7	66	62	64	Sonata Flex28	Sonata Standard	Opus2	Opus2	FSP	FSP	2	2
P8	22	1	6	Pulsar Standard	Combi40 Standard	Sonnet	Sonnet	FSP	CIS	1	0
P9	22	1	5	Combi40+ Standard	Combi40+ Standard	Opus2	Opus2	CIS	CIS	0	0
P10	56	51	52	Sonata Flex28	Sonata Flex28	Opus2	Opus2	FS4HR	FS4HR	4	4
P11	49	44	44	Sonata Standard	Sonata Standard	Sonnet	Sonnet	FSP	FSP	2	2
P12	41	36	39	Concerto Flex28	Concerto FlexSoft	Sonnet	Opus2	FS4HR	FS4HR	4	3
P13	62	55	54	Sonata Standard	Sonata Standard	Sonnet	Sonnet	FS4HR	FS4HR	4	4
P14	46	44	43	Concerto Standard	Concerto Medium	Sonnet	Sonnet	CIS	FSP	0	1
P15	49	46	46	Sonata Flex28	Sonata Standard	Sonnet	Sonnet	FSP	FSP	2	2
P16	57	55	56	Synchrony Flex28	Synchrony Flex28	Rondo	Rondo	FSP	FSP	2	2
P17	55	52	48	Sonata Standard	Sonata Flex28	Opus2	Opus2	FSP	FSP	2	1
P18	50	48	47	Sonata Standard	Sonata FlexSoft	Rondo	Rondo	FSP	FSP	2	2
P19	52	47	45	Sonata Standard	Sonata Standard	Opus2	Opus2	FS4HR	FS4HR	4	4
P20	70	63	65	Sonata Flex28	Sonata Standard	Opus2	Opus2	FSP	FSP	2	2
P21	76	71	70	Sonata Flex28	Sonata Flex28	Opus2	Opus2	FS4HR	FS4HR	4	4
P22	26	18	24	Sonata Flex28	Sonata Standard	Sonnet	Sonnet	FS4HR	FS4HR	4	4
P23	67	58	62	Concerto FlexSoft	Sonata Standard	Opus2	Sonnet	FSP	FSP	2	2
P24	50	47	40	Sonata Standard	Sonata Flex28	Opus2	Rondo	FS4HR	FS4HR	4	4
P25	50	35	36	Combi40+ Standard	Combi40+ Standard	Sonnet	Sonnet	CIS	CIS	0	0

The fitting was performed under the currently applied standard procedures for bilateral CI programming, where each device is first fit independently. Later, narrow-band noises were presented in free field for right/left loudness balancing and CI users indicated their percept of the mid-sagittal plane. Note that all CI users were very experienced with the tested coding strategy and no major changes were done on their fitting for this particular experiment.

#### Normal-Hearing Controls

Eleven NH listeners (ages 24–37 years) were enrolled in the experiments as controls. All had normal thresholds (within 20 dB of audiometric zero), as determined by a standard pure-tone audiogram (ISO 8253-1:2010). Listeners had no visual and motor disorders and were naive about the purpose of the experiments.

### Fine Structure Processing

Low-frequency sounds are coded both in place and time in the apical region of the cochlea. However, most implants only extract the envelope of the incoming signal for all electrodes (frequency bands), thus eliminating the fine-structure cues. In contrast, FSP developed by MED-EL modifies the timing of pulse stimulation to code temporal information in the low frequencies.

FSP coding is identical to the CIS-based stimulation (11), except lower-frequency channels, where channel-specific sampling sequences (CSSS) replace the fixed fast-rate pulse train. The CSSS are a series of pulses triggered by the positive zero crossings in the filter's output signals, which are then modulated in amplitude by the extracted envelope of the corresponding channel.

Based on this initial stimulation protocol, the first version of the FS coding strategy was introduced in 2006. The number of apical channels with CSSS varied from one to three according to the fitting variables for a given CI user. Later, FS4 was developed, ensuring CSSS channels up for the first four apical channels (up to 1 kHz). Finally, FS4-P was released, which allows parallel time coding on the four apical CSSS channels. In our study, and as mentioned before, we adopted our own nomenclature to define the FS coding strategy referring to the bilateral number of CSSS or FS channels (see previous section).

Normally, in bilateral CI fittings, the audio processors are not synchronized with each other. However, the apical electrodes on the FSP strategy are locked to the zero crossing of the acoustic input. Since the acoustic input is highly correlated (or “synchronized”) between ears, theoretically, the pulses can be delivered preserving ITDs. However, the sampling rate is the limiting factor for a proper zero-crossing representation. For the CSSS channels, it is typically situated between 3 and 10 kHz, which can lead to temporal accuracies of 0–333 and 0–100 μs, respectively (22). In bilateral FSP configurations, this procedure results in an interaural jitter in the order of ±100 to ±333 μs, which can perturb ITD perception.

### ILD and ITD Perception

#### Stimuli and Task

Psychometric experiments were used to measure the ILD and ITD sensitivity of the listeners, together with their potential side bias. The stimuli were generated in MATLAB (The MathWorks Inc., Natick, MA, USA), played through an external sound card (MOTU Ultralite, Cambridge, MA). For bilateral CI users, the stimuli were delivered through the CI processor's audio input (only bypassing their microphones), and NH was tested *via* headphones (Beyerdynamic, DT 770 Pro, Heilbronn, Germany). Tasks were designed as two-alternative forced choice (2AFC). After the sound was presented, the listener had to respond by pressing the left or right arrow on a keyboard, indicating the perceived side relative to the mid-sagittal plane of the head.

For ILD sensitivity testing, the level between the two inputs was changed, while the ITD was fixed at 0 s. During the test, the acoustic power of the signal was kept constant, maintaining the same overall loudness for all ILD magnitudes. This task was evaluated with two frequency ranges: low-pass (LP, 0.15–0.8 kHz), generally covering the four apical channels, and high-pass (HP, 1.5–10 kHz), where medial and basal electrodes are stimulated (electrodes 5–12). The applied ILDs for the BICI users ranged from +10 to −10 dB and were divided into 16 equal steps of 1.25 dB. Each ILD was randomly tested 15 times along the experiment, making a total of 240 trials. The NH listeners were tested over a narrower ILD range between ±5 dB (divided into 16 equal steps of 0.625 dB); in addition, the +10- and −10-dB sounds were presented to obtain the two extreme data points. Also, each ILD was evaluated 15 times, making a total of 270 trials for this group.

For ITD testing, the ILD was kept constant at 0 dB, while the onset time between the ears was systematically varied. For this task, LP (0.15–0.8 kHz) noise bursts were presented. ITDs were varied between ±2 ms for BICI users (in 16 equal 0.25-ms steps) and ±0.2 ms for NH listeners (in 16 equal 0.025-ms steps), together with +0.8 and −0.8 ms as the extreme data points. The same amount of trials as for the ILD task was performed.

#### Data Analysis

**Figure 1:**
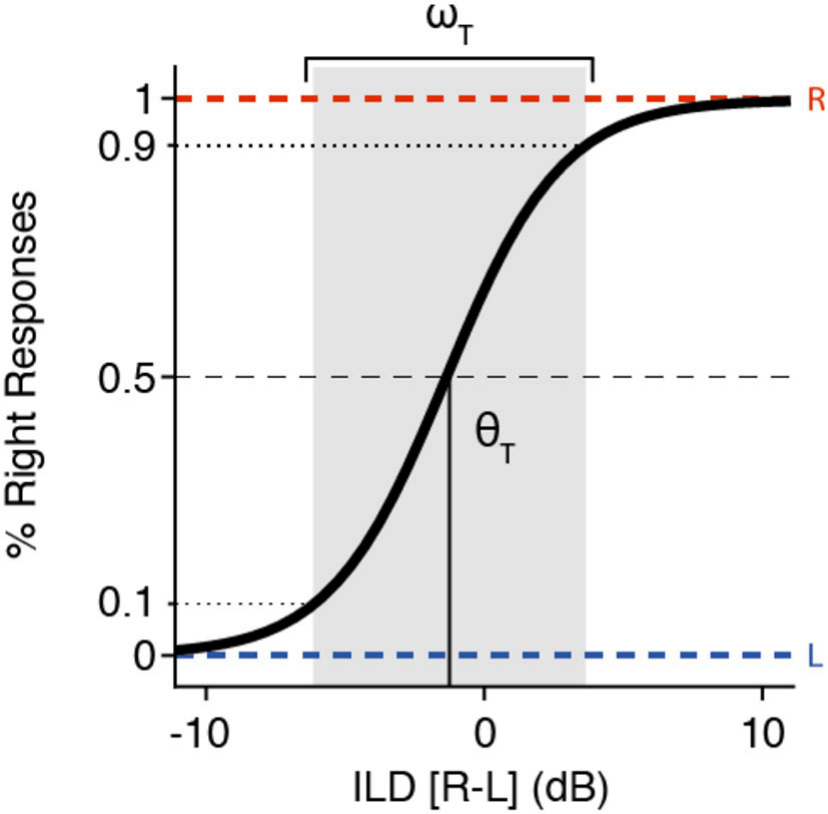
Example of an interaural level difference (ILD) psychometric curve, described by Equation (1), with its two parameters indicated. The *shaded zone* indicates the sensitivity range, ω_T_ (here, ~10 dB) around the threshold, θ_T_ (here taken at −1.25 dB).

To describe the psychophysical ILD and ITD data, we performed a sigmoid fit over the binary left/right responses with the following logistic function (23) (Figure 1):

(1) $$\begin{array}{r} p\left(x_{T}\right)=\;{\left(1+{\text{e}}^{-4.39\;\frac{\left(x_{\text{T}}-\theta_{\text{T}}\right)}{\omega_{\text{T}}}\;}\right)}^{-1} \end{array}$$

with *x*_T_ the acoustic cue of the target [ILD (in decibels) or ITD (in milliseconds)] and θ_T_ the perceptual bias (the same unit as *x*_T_). Positive/negative values reflect a left/rightward bias, and the function is point-symmetric around θ_T_ (in decibels or milliseconds). The listener's sensitivity to the cue is described by the width, ω_T_ (in decibels or milliseconds), which, in the present parametrization (Equation 1), denotes the 10–90% width of the sigmoid (see Figure 1, expressed in the same units as the stimulus). Note that the width and sensitivity are inversely related: a larger width yield, less sensitivity.

To interpret the ILD and ITD sensitivity, we extracted physiological ranges from a 5° horizontal resolution impulse response library (24). The maximum ILD/ITD value computed as a function of azimuth is used in our analysis and referred to as the physiological limit.

### Sound Localization

#### Setup and Stimuli

Sound localization performance was tested for broadband (0.15–10 kHz), high-pass (1.5–10 kHz), and low-pass (0.15–0.8 kHz) noise bursts of 150 ms. As in the psychophysical tasks, the frequency ranges were selected to cover apical (LP; channels 1–4), medial-basal (HP; channels 5–12), and the complete electrode array stimulation (BB; channels 1–12). Sound levels were presented at 50, 60, and 70 dBA and target locations were distributed over the two-dimensional frontal space, between ±75° in azimuth and ±30° in elevation. Stimuli were presented in a dark, anechoic room as described by Van Bentum et al. (25). The subjects were asked to localize these noise bursts by pointing with a rapid head movement to the perceived location of the stimulus.

#### Experiment

Each sound localization trial started with the presentation of a green fixation LED at straight ahead (0° azimuth and 0° elevation). Using a head-fixed laser pointer, the subjects were instructed to align the laser dot with the fixation LED to ensure the same head orientation at the start of each trial. After the subject pressed a button, the fixation light was turned off within 100–300 ms, followed by the target sound with a 200-ms delay. The subjects were asked to point the laser dot as fast and accurately as possible toward the perceived sound location. The acquisition time of the head movement was 2.5 s.

#### Data Analysis

Head movements were detected automatically from the calibrated head position signals using a custom-made Matlab script that checked for head velocities exceeding 20°/s. Onset and offset of the head movements were detected by the program and visually checked off-line.

**Figure 2:**
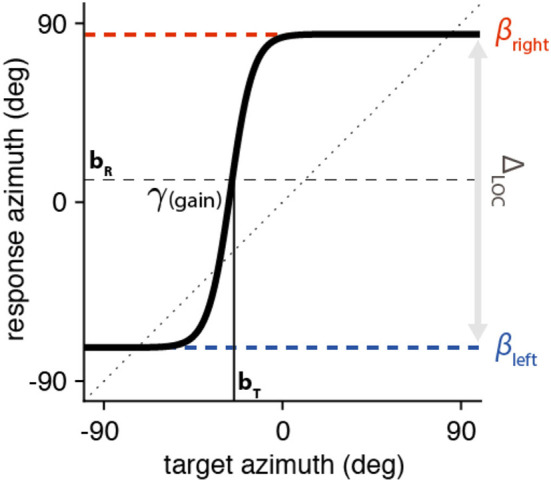
Sound localization fitting example as described by Equation (3). The sigmoid is centered around the target where the curve is equidistant from the two asymptotes (in the example, *b*_T_ = −24° (leading to a rightward response bias) and *b*_R_ = +9°, respectively). The maximal localization gain, or γ, is calculated at the target bias location (α_*T*_ = *b*_*T*_; in this example, γ = 4.9). The asymptotes (β_left_ = −73° and β_right_ = +84°) define the range of the localization response (Δ_LOC_ = 157°).

The target–response relationship of the BICI users was modeled with a sigmoid (Figure 2) using the following equation:

(2) $$\begin{array}{r} \alpha_{R}=90\cdot c\cdot \tanh\left(\frac{g\cdot \;\left(\alpha_{T}-b_{T}\right)}{90}\right)+b_{R} \end{array}$$

α_*T*_ and α_*R*_ correspond to the target and response angles (in degrees), respectively. The range (Δ_LOC_), or compression, of the localization response is quantified by *c* (dimensionless), e.g., if *c* is 0.5, the responses are constrained between ±45° in azimuth. The slope of the sigmoid is determined by c and *g* (dimensionless). Finally, parameters *b*_T_ and *b*_R_ correspond to the target and response biases (in degrees), respectively. The first derivative of the sigmoid at α_*T*_ = *b*_*T*_ provides the maximum slope of the fit (i.e., the maximum localization gain), γ, which is an interaction of the compression of the response and the sigmoid gain:

(3) $$\begin{array}{r} \gamma \;=c\;\cdot \;g \end{array}$$

The limits of the response range (in degrees) are determined by the asymptotes of the fit and are referred to as β_left_ = *b*_r_ – 90*c* and β_right_ = *b*_r_ + 90*c*, for the leftward and rightward limits, respectively. Note that a perfect localization response would result in γ= 1 and *b*_T_ = *b*_R_ = 0°, with β_left_ ≤ 90° and β_right_ > 90°. A gain that far exceeds 1 (γ >> 1) suggests a tendency toward left/right discrimination (what we here refer to as lateralization performance); in an extreme case, the sigmoid fit would resemble a step function, showing only left/right localization (extreme lateralization).

To obtain an overall measure for the response accuracy, we also computed the mean absolute error (MAE) across trials, according to:

(4) $$\begin{array}{r} MAE=\;\frac{1}{N}\;\overset{N}{\underset{n=1}{\sum }}|\alpha_{\text{R}}^{n}-\alpha_{\text{T}}^{n}| \end{array}$$

with α_*R*_ the response azimuth (in degrees), α_*T*_ the target azimuth (in degrees), and *N* the number of trials. Note that, in our experiments and setup configuration, extreme lateralization performance (i.e., if α_*T*_ ≥ 0°, α_*R*_ = +75°, and for α_*T*_ < 0°, α_*R*_ = −75°), would result in a MAE = 37°.

### Statistical Analysis

For sound localization analysis, separate *N*-way ANOVAs were performed with as independent factors—subject (random), presentation level, stimulus bandwidth, and FSP group—and as dependent variables—*b*_T_ (target bias), *b*_R_ (response bias), *c* (compression factor of the response range), γ (gain), and MAE.

Means and the 95% confidence intervals of the dependent variables are also reported in the *Results* (±2 times the standard error of the mean). This confidence interval was also used to calculate the statistical significance between means. All analyses were performed with MATLAB software.

## Results

### ILD and ITD Perception

**Figure 3:**
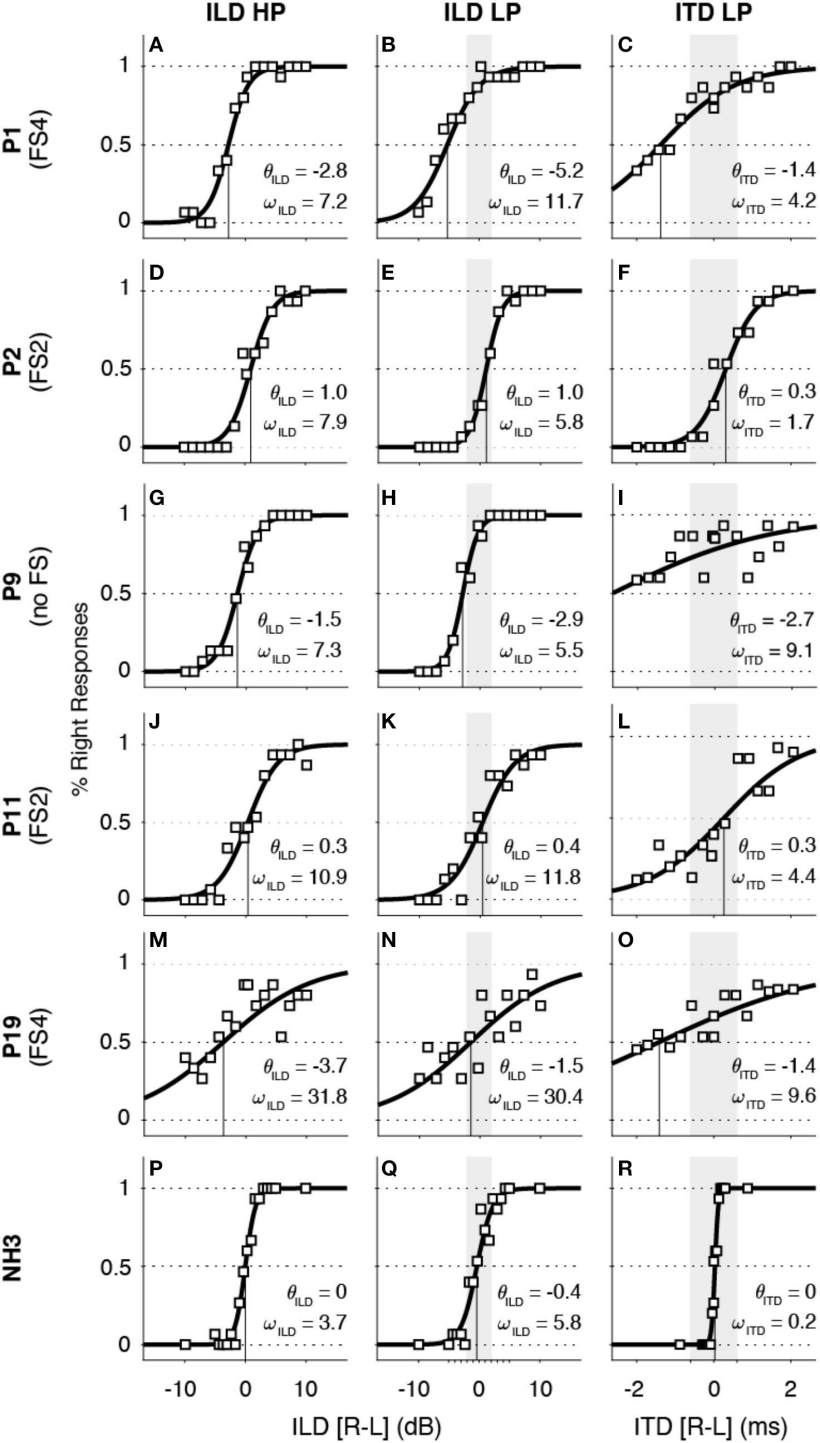
Interaural level difference (ILD, *left* and *middle column*s) and interaural timing difference (ITD, *right column*) data and sigmoid fits for four representative examples of BICI listeners **(A**–**O)** and one normal-hearing (NH) control **(P**–**R)**. In all ITD panels, the *gray area* indicates the physiological range for ILD low-pass (LP, ±2 dB) and ITD LP (±600 μs) that is within the stimulus intensity range. Note that four out of five CI-users demonstrate some variable ITD sensitivity within the physiological range and that all show ILD high-pass (HP) sensitivity.

To determine the sensitivity of ILDs and ITDs, we use ω as a measure of cue sensitivity and θ to quantify the right/left bias (see *Methods*). The stimuli were LP and HP noises, addressing the apical and medial-basal electrodes for CI users, respectively. In general, FSP cochlear implant listeners revealed some, albeit poor, ITD sensitivity. All subjects had their sensitivity range (ω) beyond the physiological range (see gray area in Figure 3), where this cue will saturate under free-field hearing. However, most (*n* = 22 out of 25) participants were able to detect high-frequency ILDs within a usable range (<20 dB). Both tasks showed considerable variability among the CI subjects in sensitivity (ω) and threshold (θ).

To illustrate the data analysis and responses for individual cases, we show the results from five representative BICI listeners with zero, two, and four FSP channels (Figures 3A–**O**) as well as for a NH control listener, for comparison (Figures 3P–**R**). The sensitivity of the ITD cue for the BICI users ranged from 1.7 ms (FS2 listener P2; Figure 3F) to 9.6 ms (no FS listener P19; Figure 3L), while the NH example showed a ω_ITD_ that was much smaller, at 0.2 ms (NH3; Figure 3O). Although the modest sensitivity to ITD is insufficient to correctly lateralize the sound, it may nevertheless have offered a weakly informative cue for the listeners to be perceived (e.g., see Figures 3F,L).

In contrast, the ILD sensitivity for the BICI users and the NH example, quantified by ω_ILD_, were more comparable. While P19 performed more poorly with a sensitivity of ω_ILD_ = 31.8 dB for HP and ω_ILD_ = 30.4 dB for LP, listener P2 yielded ω_ILD_ = 7.9 dB and ω_ILD_ = 5.8 dB for HP and LP sounds, respectively. These examples illustrate the large variability across listeners, but indicate also that most of them had a well-defined sensitivity to this cue. The threshold value, θ_ILD_, which characterizes the balance between the right and left ears, varied across listeners. Note that the LP value for θ_ILD_ is correlated to θ_ITD_ as the ITD was measured at 0 dB ILD for the same frequency range for all CI listeners (*r*^2^ = 0.59, *p* < 0.001). For example, P1 had a rightward ILD bias for LP stimuli with θ_ILD_ = −5.2 dB and also a negative ITD bias of θ_ITD_ = −1.4 ms for the same sounds. Also, listeners P9 and P19 showed a consistent relation between the LP-ILD and ITD tasks, but although they yielded smaller ILD thresholds than P1, their ITD thresholds were more extreme to the right than for P1.

**Figure 4:**
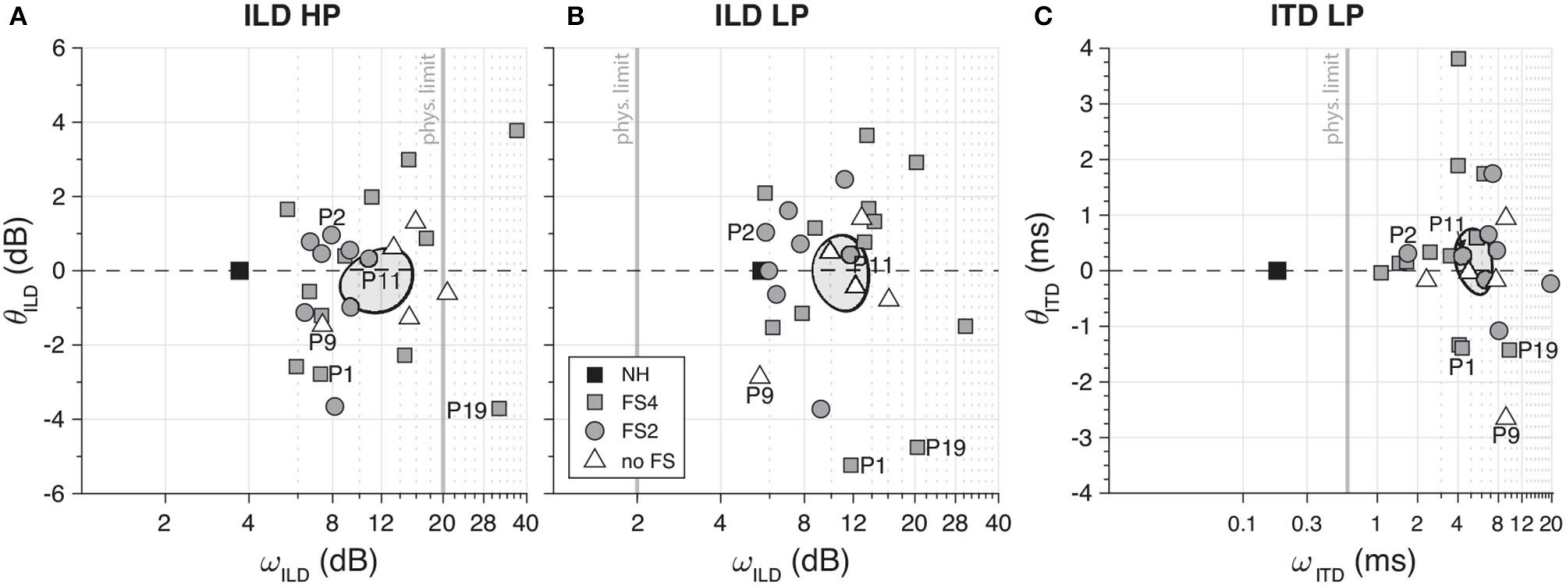
Overall bias (θ) and sensitivity (ω) of interaural level difference (ILD) high-pass **(A)** and low-pass **(B)** and interaural timing difference (ITD) low-pass **(C)**. *Vertical gray lines* represent the cue's physiological range values. In all panels, the mean and variability (95% confidence interval) of the data of BICI users are represented by *ellipses*. As reference, the mean of the NH results is marked with a *gray square*. Note that for ILD and ITD low-pass, all CI users fell outside the boundaries (2 dB and 600 μs, respectively) indicated by the physiological range. Normal-hearing (NH) controls, however, demonstrated clear ITD LP perception.

An overview of the sensitivity (ω) and bias (θ) for all sound types and subgroups is provided in Figure 4. The vertical gray line in Figures 4A–**C** illustrates the maximum ILD/ITD for each bandwidth (see *Methods*). We did not observe an effect of the different FS subgroups on ILD or ITD sensitivity (confidence intervals of the means were overlapping). When pooling all groups to compute the overall mean (gray shadowed area in Figure 4), all BICI users yielded ω_ITD_ beyond the physiological limit (mean of 5.5 ± 1.7 ms; Figure 4C), which reflects the difficulty to use ITD cues in the free field. Note, however, that some FS subjects demonstrated better ITD sensitivity (around 1 ms), which is not far from the physiological limit and, although not sufficient for veridical lateralization, could still serve as a weak but informative cue for azimuth (e.g., P2; Figure 3F). Similarly, the sensitivity for low-frequency ILDs fell outside its small physiological range (±2 dB) for all CI listeners (mean = 11.2 ± 2.6 dB; Figure 4B). However, most listeners demonstrated ILD sensitivity for HP (mean = 11.9 ± 3.5 dB; Figure 4A) and LP sounds (mean = 11.2 ± 2.6 dB; Figure 4B). All normal-hearing controls showed a high sensitivity to ILD in HP (mean of 3.7 ± 1.7 dB; Figure 4A) and ITD in LP (mean of 0.12 ± 0.02 ms; Figure 4C). It is noteworthy that, in normal hearing subjects, the reduced sensitivity to weak low-pass ILDs is overcome with the ITD perception. There was considerable variability in the biases across BICI listeners, which could reflect that one device was programmed slightly louder than the other. Although the overall means for θ_ILD_ (−0.2 ± 0.9 dB for HP and −0.1 ± 1.0 for LP; Figures 4A,B, respectively) and θ_ITD_ (0.2 ± 0.6 ms; Figure 4C) did not differ from zero, some subjects had a clear right or left tendency. All NH listeners showed balanced responses for ILD (0 ± 0.3 dB for HP and 0 ± 0.2 for LP; Figures 4A,B, respectively) and ITD (0.10 ± 0.02 ms; Figure 4C), as expected.

### Sound Localization

Localization performance in the free anechoic field was tested with BB, HP, and LP sounds presented in pseudorandom order at one of the three presentation levels (50, 60, or 70 dB SPL) in the two-dimensional frontal hemifield. The presentation levels did not affect the response gain, γ (*F*_*df*__= 2_ = 0.8, *p* = 0.5), the perceived target range, *c* (*F*_*df*__= 2_ = 1.9, *p* = 0.1), target bias, *b*_T_ (*F*_*df*__= 2_ = 1.6, *p* = 0.2), or response bias, *b*_R_ (*F*_*df*__= 2_ = 1.1, *p* = 0.3). Therefore, we pooled all data across levels and analyzed the differences on the three stimulus bandwidths.

**Figure 5:**
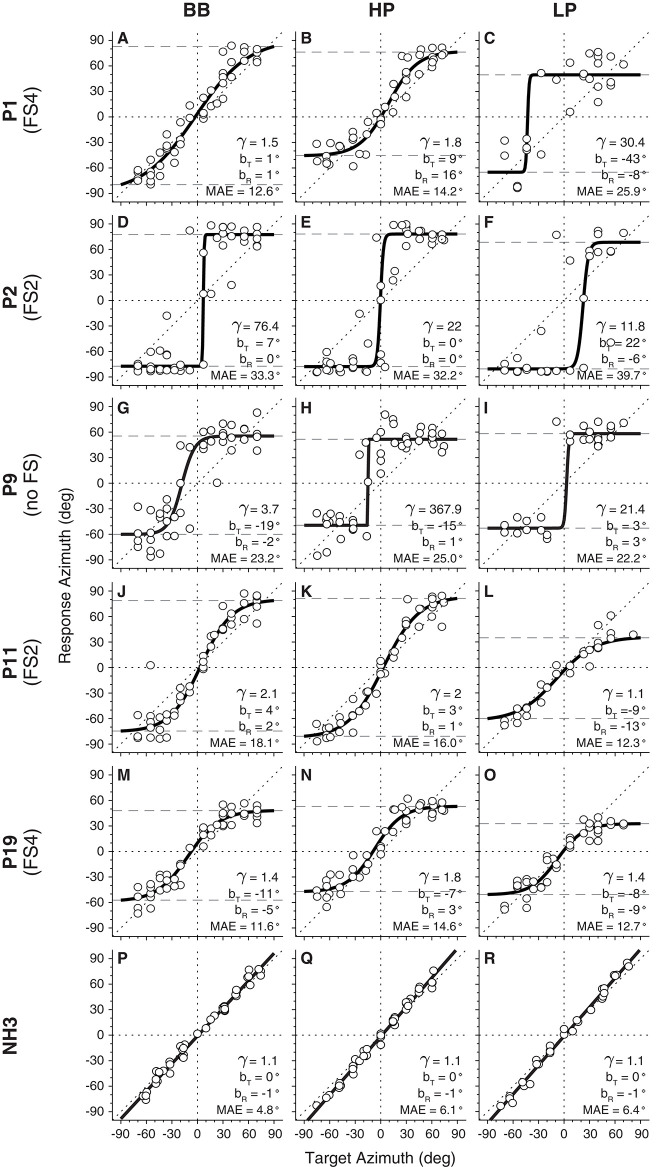
Target–response relationship of horizontal sound localization for broadband (BB), high-pass (HP), and low-pass (LP) stimuli. Five examples of BICI listeners **(A**–**O)** are presented, illustrating the overall performance of the group. A normal-hearing (NH) control listener **(P**–**R)** is shown as reference.

To illustrate the overall type of responses from the BICI listeners and the sigmoid fit analysis, Figure 5 shows the results of the same subjects as on the ILD/ITD tasks. Subject P1 localized BB sounds with a good near-linear stimulus–response relation with γ = 1.5 and practically no bias (*b*_R_ = 1.0° and *b*_T_ = 1.0°; Figure 5A). Moreover, the difference in the BB and HP performance (Figures 5A,B) suggests that the subtle low-frequency cues may have been useful and beneficial for this listener. However, the localization performance for LP sounds yielded noisier responses with a higher gain (γ = 30.4), a higher MAE (25.9°), and a pronounced rightward bias (*t* = −43°; Figure 5C).

Listener P2 is an extreme example of sound lateralization as the responses were directed to the far left and far right, irrespective of the stimulus type presented (Figures 5D–**F**). This listener had a clear leftward bias for the LP stimuli, in line with a positive target bias (*b*_T_ = 22°). The extreme lateralization performance yielded high MAEs (mean MAE = 35° for the three sound types, which is closer to the actual theoretical lateralization value of 37°). P9 generated responses to fixed locations, around ±60°, in the left and right hemifields (Figures 5H,I). In this case, the responses were slightly more variable, but with MAEs that were substantially smaller than for P2.

Listeners P11 and P19 showed similar systematic stimulus–response relations for azimuth than did P1. In both cases, the central range (±40°) showed an almost linear target–response relationship, but saturating at the edges. Interestingly, both listeners also yielded systematic localization responses for the LP stimuli, with small MAEs and near-normal gains, albeit with a reduced response range (Δ_LOC_ = ~90–95°; Figures 5L,O).

**Figure 6:**
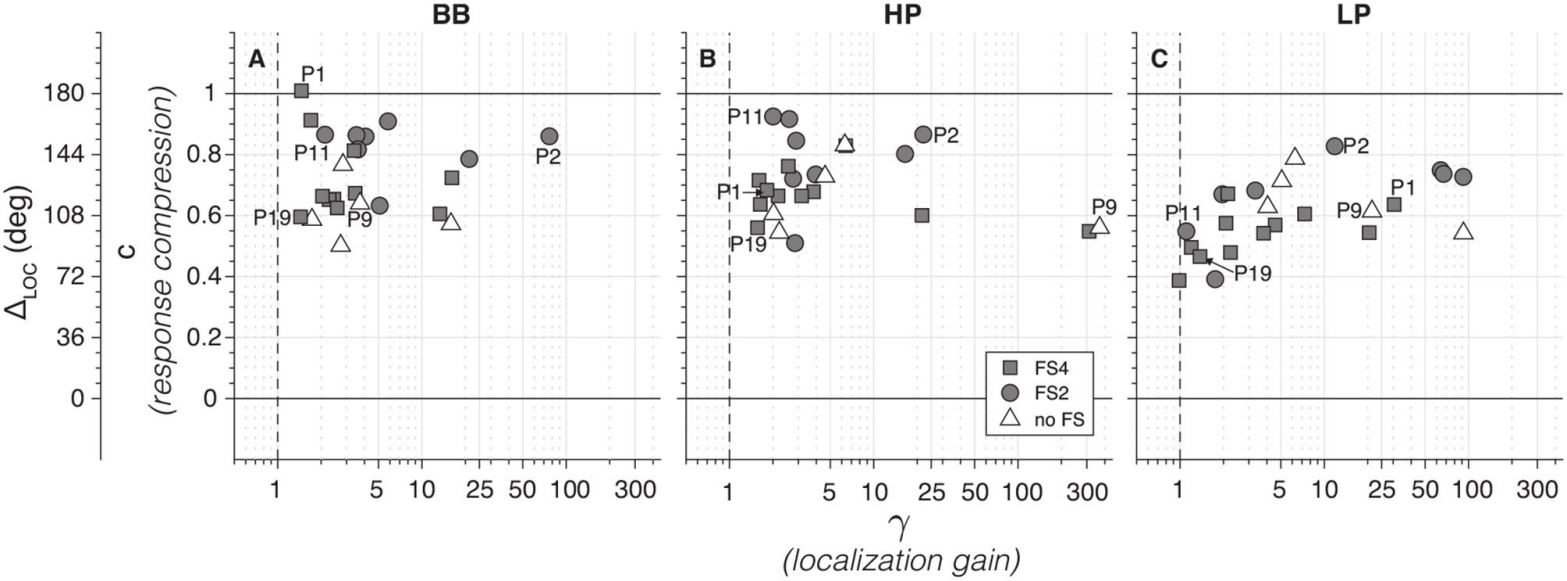
BICI sound localization gain (γ) compared to the response compression (*c*) for broadband **(A)**, high-pass **(B)**, and low-pass **(C)**. Note that a perfect sound localization will include a γ = 1 and *c* ≥ 1. For an easier interpretation, a secondary *y*-axis with the Δ_LOC_ is also shown. Note that a subgroup of 11 fine-structure processing (FSP) listeners show gains <4 for low-pass (LP) sounds, indicative of localization (**C**; cf. Figure 5). In contrast, all no-FSP listeners have higher gains, which indicates lateralization behavior.

To quantify the sound localization performance of all listeners, Figure 6 shows the localization gain (γ) against the response compression (*c*, also represented as Δ_LOC_). Due to the lack of significant differences on γ (*F*_*df*__= 2_ = 0.49, *p* = 0.6) and *c* (*F*_*df*__= 2_ = 0.59, *p* = 0.559) between the three groups, we calculated the overall mean per CI group for each stimulus type. Generally, BICI listeners yielded localization gains >1 for BB sounds (mean = 8.3 ± 6.5, median = 3.4; Figure 6A), for HP stimuli (mean = 33 ± 40, median = 2.8; Figure 6B), and for LP noise (mean = 19.3 ± 12.7, median = 4.5; Figure 6C). Interestingly, only listeners with FSP showed gains γ <4 (11/19) for the LP stimuli (Figure 6C), while all five “no FS” listeners yielded higher gains. This suggests that some BICI listeners appeared to benefit from the weak yet informative low-frequency localization cues provided by the FSP protocol. Note also that the response range was reduced for all BICI subjects as the response compression, *c*, was significantly below 1, with means of 0.73 ± 0.06 for BB (Figure 6A), 0.70 ± 0.05 for HP (Figure 6B), and 0.60 ± 0.05 for LP sounds (Figure 6C). We also observed a stronger reduction of the response range for LP sounds (mean = 109 ± 9°) when compared to the BB (mean = 130 ± 9°) and HP (mean = 126 ± 9°) stimuli.

**Figure 7:**
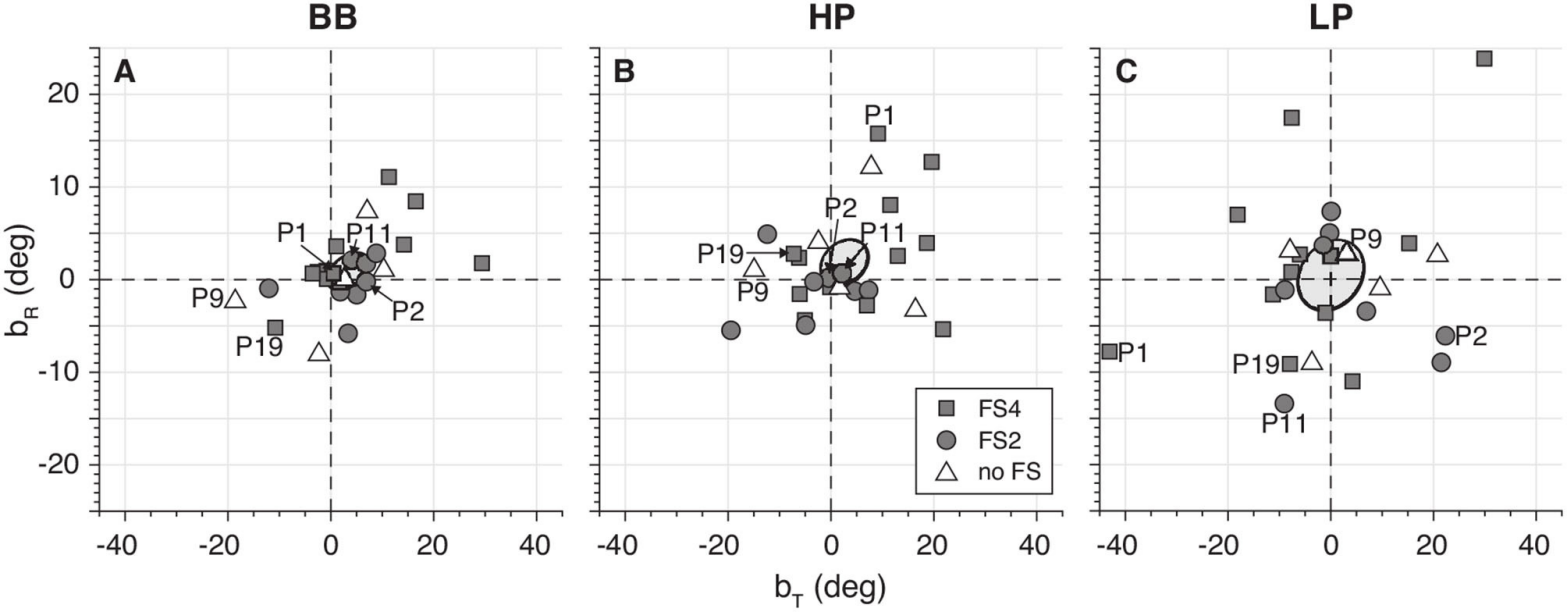
Target (*b*_T_) and response (*b*_R_) bias for broadband **(A)**, high-pass **(B)**, and low-pass **(C)**. Note the considerable variability in these measures across listeners.

Figure 7 shows the results for the localization stimulus and response biases. Although the overall mean bias for BB (*b*_T_ = 3.4 ± 4.2°; *b*_R_ = 0.9 ± 1.8°), HP (*b*_T_ = 2.6 ± 4.6°; *b*_R_ = 1.8 ± 2.4°), and LP (*b*_T_ = 0.02 ± 6.5°; *b*_R_ = 0.4 ± 3.7°) did not differ from zero, there was considerable variability across participants, evidencing some remaining perceptual asymmetries between sides.

**Figure 8:**
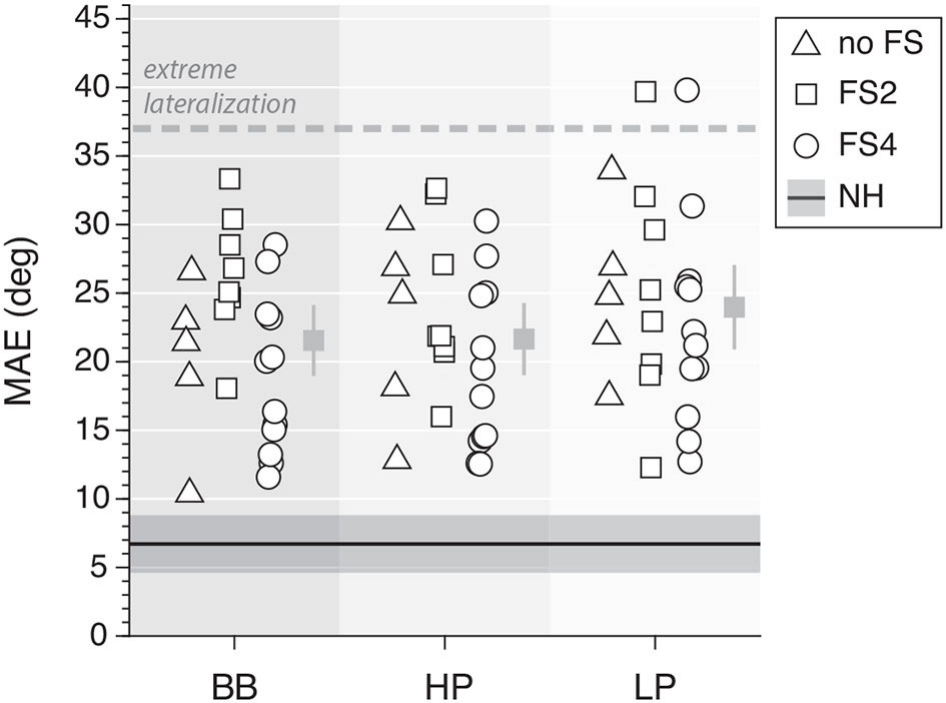
Mean absolute error of sound localization performance for all frequency ranges. *The dashed line* indicates the theoretical extreme for a pure lateralizer and the *dark solid line* represents the mean and 95% confidence interval for the normal-hearing controls. The mean and its 95% confidence interval for CI users are indicated per type of noise (*gray squares*).

As an overall measure for the localization performance of the bilateral CI groups, we computed the MAE for the different sounds (Figure 8). Overall, the MAE of the CI listeners was around 15° higher than the NH performance, but performance was much better than for pure lateralization. BB and HP yielded a similar result, with 21 ± 3° and 22 ± 3°, respectively. LP sounds yielded slightly higher MAEs, with a mean of 24 ± 3°. The NH controls performed with low MAEs across frequencies, with an overall mean of 7 ± 1°.

### ILD and Free-Field Sound Localization Bias

**Figure 9:**
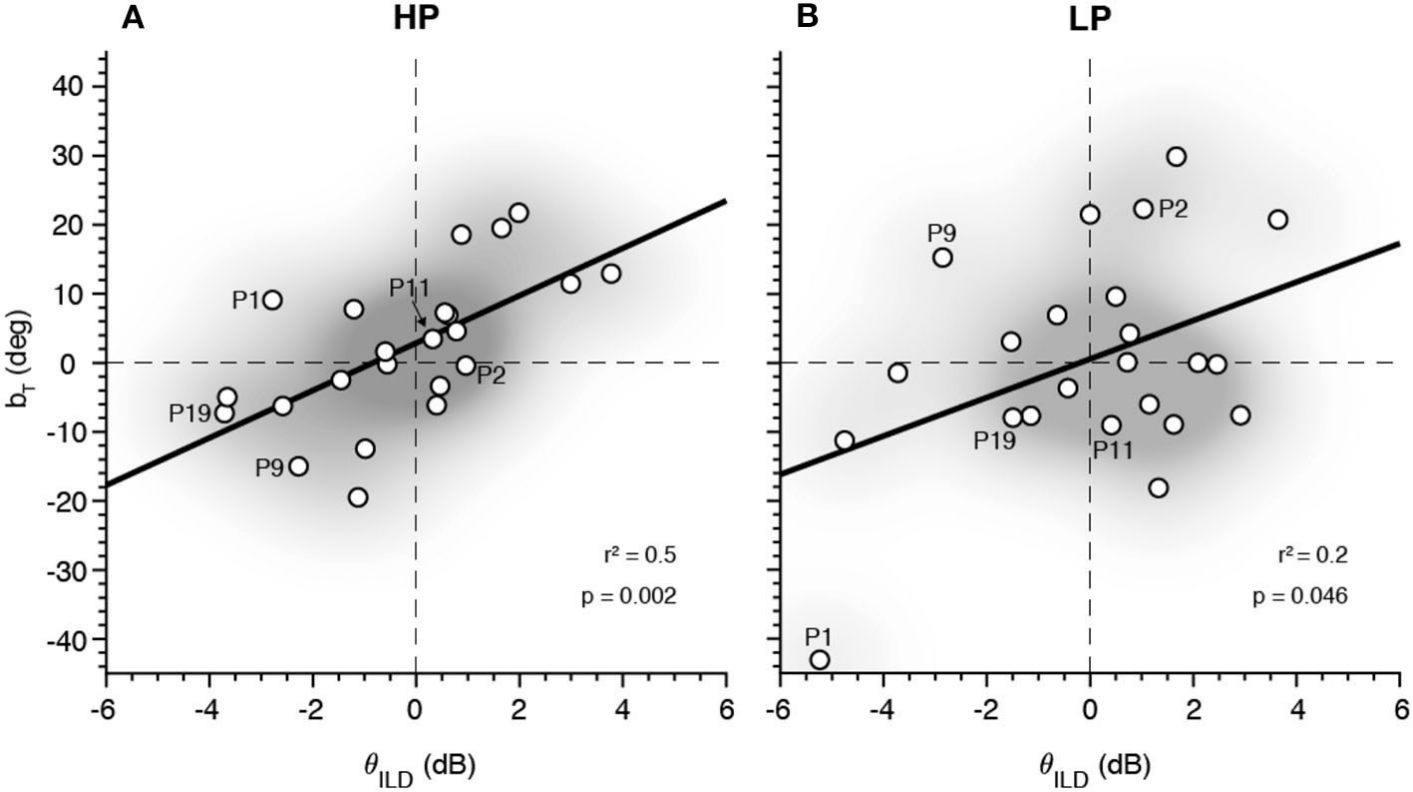
Relation between the interaural level difference (ILD) (θ_ILD_) and the target sound localization bias (*b*_T_) for high-pass (HP) **(A)** and low-pass (LP) **(B)**. The distribution density of the data points is illustrated with the *shadowed gray area*.

The left/right bias was quantified for both psychophysical experiments (as θ for the ILD/ITD tasks and *b*_T, R_ for sound localization, respectively). As representative measures, we here present θ_ILD_ and *b*_T_ for the HP and LP stimuli. We wondered whether the right or leftward free-field target localization bias, *b*_T_, could be predicted from the ILD psychophysical threshold, θ_ILD_ (Figure 9). We found that, for both sound types, the slope of the regression was positive and significantly different from zero (*p*_HP_ = 0.001 and *p*_LP_ = 0.046, for HP and LP sounds, respectively). Although the coefficients of determination were not high ($r_{\text{HP}}^{2}$ = 0.4 and $r_{\text{LP}}^{2}$ = 0.2), it is a considerable correlation given that both parameters are from different fitting models. Furthermore, the fitting direction was as expected as well as a higher correlation for HP, where the cue sensitivity is better defined.

## Discussion

As shown in the presented study, many bilateral CI users have a remarkably good localization performance, which is mainly attributed to adequate ILD processing. However, we also provided evidence that some listeners may have had access to rudimentary ITD information with but also without the FSP strategy. Furthermore, there was a large variability in performance, which so far remains unexplained. Based on our results, we argue that our data suggest that CI users may learn to successfully integrate even rudimentary binaural information.

### ILD and ITD Perception

The bilateral CI listeners in our study were mostly sensitive to ILDs (Figure 4A) since all demonstrated a good sensitivity to cues for high-frequency ILDs. Furthermore, our results show that some listeners had a weak ITD perception within the physiological range (as would be generated in the free field). Some CI listeners with FSP encoding demonstrated a monotonic stimulus–response relation for LP sounds (e.g., Figures 5L,O). This suggests that exposure to weakly informative but robust and consistent cues in everyday life might prompt CI listeners to learn to localize even low-frequency sounds (26). Our data support the recent study by Elköf and Tidelhom (21), who reported that half of their FSP population had ITD perception within the physiological range. The participants in their group were all of young age (8–13 years) and had been implanted bilaterally prior to their third year of age. As the brain is most plastic during the early stages in life (27–29), these young listeners may have been able to exploit the information provided from FSP stimulation and use the ITDs also in free-field listening. In the present study, only two of the adult listeners (22 years each) had been implanted at a young age (P8 and P9: 1 year old on the right side), but with a 4-year implantation gap for the second, left ear, and without the FSP encoding protocol in their devices.

However, several studies have demonstrated a life-long plasticity in the human auditory system, which might help CI listeners with post-lingual deafness. Yet, the localization cues provided by the CIs should be unique and consistent for any source location as inconsistent and ambiguous cues cannot induce successful perceptual learning (26, 30–34). The observation that some of the FSP listeners had learned to exploit the subtle and poor ITD cues provided by the FSP encoding protocol may be a promising approach for future improvements.

Lack of ITD perception may also be related to the neural health of the cochlea. In other words, potentially, ITD perception may be reached in CI users with substantial neural substrate of the cochlear and spiral ganglion at the lower-frequency region. In addition, ITD perception may also be influenced by auditory central processing capabilities. In the presence of residual hearing at lower frequencies, the residual function of the peripheral neural substrate can be assessed objectively by measuring auditory steady-state response (ASSR) on each side, and central processing skills may be assessed using binaural masking level difference (BMLD) as an objective measure of binaural cue integration. Due to the lack of residual hearing in our subjects, these measurements would not have been possible to perform in this study group. However, since at present candidates often have considerable residual hearing pre-implantation, future prospective studies may include psycho-acoustic and objective measures to assess the auditory pathway prior to bilateral implantation. This would expand insights into the variables that determine spatial hearing.

### Sound Localization

The overall sound localization performance is in accordance with other results reported for bilateral CI listeners (1, 5, 6, 20), albeit that accuracy and precision remained worse than that of normal-hearing listeners (Figure 8). Furthermore, we observed some differences in the free-field sound localization performance between FS and no-FS listeners as 11 out of 19 FS listeners had localization gains <4 for low-pass sounds (Figure 6C). However, this evidence is not strong enough to support the hypothesis that FS listeners have better localization at a group level.

The deprived ITD sensitivity and the poor ILD representation might underlie this impoverished sound localization performance. Moreover, the weakness of the low-frequency free-field cues seems to be reflected in spatially compressed localization responses (Δ_LOC_ <180°; Figure 6C). Still, we observed a very good sound localization performance for LP stimuli in some participants (e.g., P11; Figure 5L). However, considering the MAE values of each group and each frequency band, no significant difference was found (Figure 8).

### The Effect of CI Asymmetry

In bilateral CI users, asymmetry is quite common due to a mismatch of the bilateral electrode's position or a difference with regard to the time of implantation between the two sides. While one might argue that these asymmetrical factors might affect the listener's spatial hearing performance, our data did not show any strong supportive correlation for this association. Although the exact position of the electrode array and its insertion angle was not measured, since the same electrode design and length were used on each side and with reported full insertion of all electrodes, a comparable insertion angle between both ears is assumed. However, the variation in response bias for the ILD task and the sound localization task might indicate that bilateral CI fitting was not always well-balanced between ears (Figures 4, 7). Typically, each side was fitted independently, and the perception of equal loudness between sides was subjectively assessed in the clinic. However, a subjective procedure may have led to idiosyncratic unbalanced hearing shown in the free-field sound localization results as idiosyncratic non-zero bias. Up to now, there is no standardized protocol in the CI fitting software that helps audiologists to correctly balance each ear according to the subject's perception. In our study, we see that a simple ILD task with a 2AFC design (left/right response) could be a good initial predictor of the free-field localization bias. However, even with perfectly balanced CIs, sounds might still not be fused as one single source due to device- and patient-related issues (35). Potential asymmetry between left/right electrodes, as well as between-ear frequency allocation tables could also perturb the frequency-specific binaural integration channels (36, 37).

Clearly, one might not expect that CI listeners, equipped with a restricted number of frequency channels and a highly limited dynamic range, can approach similar spatial resolution and localization performance as normal-hearing listeners, who can precisely process the encoded information from over 3,000 channels over a huge dynamic range. Yet, binaural integration in bilateral CI recipients might be further improved in the future with optimized bilateral encoding strategies that allow a better synchronization between the two devices, an optimized spectral overlap, and a reliably balanced loudness perception. Moreover, training for spatial hearing is normally not part of CI standard of care, but should be considered as this rehabilitation approach might improve spatial hearing skills in CI users. This way, CI users may truly exploit spatial auditory cues and might map them into a veridical representation of the acoustic environment.

## Data Availability Statement

The raw data supporting the conclusions of this article will be made available by the authors, without undue reservation.

## Ethics Statement

The studies involving human participants were reviewed and approved by ENT clinic of St. Elisabeth-Hospital of the Ruhr-University in Bochum, Germany. The patients/participants provided their written informed consent to participate in this study.

## Author Contributions

SA, MA, and SB designed and performed the experiments. SA analyzed the data and wrote the paper. AE, CV, SB, SD, and JT supported the data collection and provided critical revision of the paper. AV, AS, MA, and EM supervised the findings and wrote the final manuscript. EM was the initiator of this collaborative study. All authors contributed to the article and approved the submitted version.

## Conflict of Interest

SB is an employee of MED-EL. The remaining authors declare that the research was conducted in the absence of any commercial or financial relationships that could be construed as a potential conflict of interest.
